# Etiological factors commonly related to the need of endodontic treatment in individuals with orofacial clefts

**DOI:** 10.4317/jced.57980

**Published:** 2021-06-01

**Authors:** Viviane-da Silva Siqueira, José-Francisco Mateo-Castillo, Lidiane-de Castro Pinto, Daniela Garib, Claudia-Ramos Pinheiro

**Affiliations:** 1 Endodontics Sector, Dentistry Department, Hospital for Rehabilitation of Craniofacial Anomalies, University of São Paulo (HRAC/ USP), Bauru, São Paulo, Brazil; 2 Orthodontics Sector, Dentistry Department, Hospital for Rehabilitation of Craniofacial Anomalies, University of São Paulo (HRAC/ USP), Bauru, São Paulo, Brazil; 3 Endodontics Sector, Dentistry Department, Specialization in Endodontics, Postgraduate Center for Dentistry - CPO Uningá, Bauru, São Paulo, Brazil

## Abstract

**Background:**

Dental treatment is fundamental in the rehabilitation of individuals with orofacial clefts, due to their oral condition; when indicated, endodontic therapy allows elimination of infection of the root canal system. Aim: To analyze, by a retrospective study, the most prevalent type of orofacial cleft, the etiological factors most commonly related to the endodontic treatment need, as well as their success and failure rates.

**Material and Methods:**

This study analyzed data from 136 records (76 females and 60 males) with mean age of 19 years and 7 months, who met the inclusion criteria. Data were collected including the type of cleft, etiological factors that led to the need of endodontic treatment, as well as their success and failure rates. The statistical analysis was performed by the chi-square, Kruskal-Wallis, Fisher’s Exact and Batista Pike tests.

**Results:**

Among the etiological factors, were pulp involvement due to caries, endodontic treatment for prosthetic rehabilitation, tooth resorptions, for orthodontic movement, dental trauma and indication of internal tooth bleaching; the most prevalent factor was pulp involvement due to caries. Among all data analyzed, cleft lip and palate presented the highest percentage, and there was predominance of treatment success compared to failure.

**Conclusions:**

The well-conducted root canal treatment is necessary for dental rehabilitation, maintaining the masticatory function and esthetic harmony of these individuals.

** Key words:**Cleft lip, Cleft palate, Endodontics.

## Introduction

Dental treatment is fundamental for the rehabilitation of individuals with cleft lip and palate. These patients presented greater difficulty in oral hygiene due to their oral condition, such as tooth crowding, malpositioned teeth and supernumerary teeth. The poor oral hygiene leads to the occurrence of dental caries; if no intervention is performed, clinical studies demonstrate that the cariogenic bacteria and their byproducts may reach the dental pulp, leading to the need of endodontic therapy (1-3).

The absence of pain, edema, fistula, the reestablishment of masticatory functions and occlusion, and reduction of the periapical lesion observed radiographically with areas with formation of new bone, would be sufficient parameters to determine the success of endodontic therapy. However, the pain threshold varies across individuals, periapical radiographs may present distortions and provide only bidimensional images (4). Therefore, the individual should be informed about the importance of follow-up, which should be performed yearly, for up to four years after completion of endodontic treatment (5,6).

Therefore, it is interesting to know, by a retrospective study, the etiological factors most commonly related to the need of endodontic treatment, as well as the predominant types of orofacial clefts and the success and failure rates of endodontic treatments, thus allowing quality control of the service delivered, aiming to review the efficiency of practices.

## Material and Methods

The study was approved by the Institutional Review Board of a Brazilian tertiary cleft center under protocol n. 11586919.0.0000.5441. The study involved initial screening of the records of 400 individuals with non-syndromic cleft lip (CL), cleft palate (CP) and cleft lip and palate (CLP), aged 15 to 25 years. The following inclusion criteria were established: patients submitted to endodontic treatments finalized in the last ten years who attended the clinic for clinical and radiographic follow-up, for establishment of the criteria of success and failure of endodontic therapy performed. Thus, data were collected from 136 records (76 females and 60 males) with mean age 19 years and 7 months.

The study analyzed and quantified data from the patient records concerning the types of clefts, etiological factors that led to the need of endodontic treatment, which were divided into five groups. The study also evaluated the treatment success and failure rates, when the patient returned for scheduled follow-up on the recommended timing (one to four years, depending on the previous pulp diagnosis).

Distribution of the type of cleft in the different groups was assessed by the chi-square and Kruskal-Wallis statistical tests. Comparisons between groups (etiological factors) were performed by statistical analysis using the Fisher’s exact test, and the success and failure rates were analyzed by the Batista Pike test.

## Results

In the presented results, it should be highlighted that, regardless of the etiological factor, the endodontic treatment was indicated and performed in all cases. According to the data obtained, the etiological factors were subdivided into: Group I, individuals with pulp involvement due to caries; Grupo II, root canal treatment for prosthetic purposes; Group III, related to tooth resorptions; Group IV, individuals submitted to orthodontic movement; and Group V, endodontic therapy performed for other reasons, such as dental trauma and indication for internal tooth bleaching.

**Figure 1:**
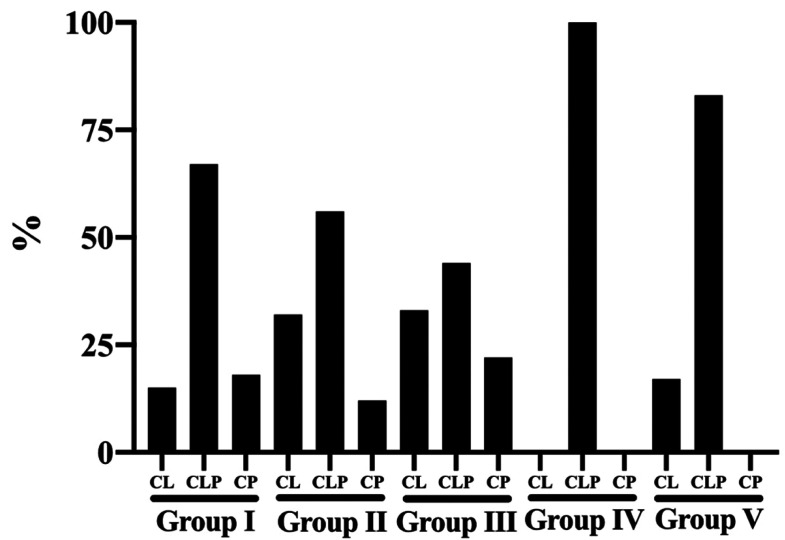
Distribution of groups according to the type of orofacial cleft.

Figure 1 evidences the distribution of groups according to the type of orofacial cleft, including cleft lip (CL), cleft lip and palate (CLP) and cleft palate (CP). Regardless of the etiological factor for the need of endodontic treatment, cleft lip and palate was the most prevalent. The following percentages were observed: Group I – CLP 66.7%, CP 17.9% and CL 15.4%; Group II – FLP 56.1%, CL 31.7% and CP 12.2%; Group III – CLP 44.5%, CL 33.3% and CP 22.2%; Group IV – CLP 100%; and Group V – CLP 83.3% and CL 16.7%.

Table 1 presents the success and failure rates of endodontic treatments according to the type of cleft, evidencing that, regardless of the type of orofacial cleft, the endodontic treatments were successful. Comparison between types of clefts, taking cleft palate (CP) as reference, evidenced statistically significant difference between cleft palate (CP) and cleft lip (CL), *P*=0.01.

**Table 1:**
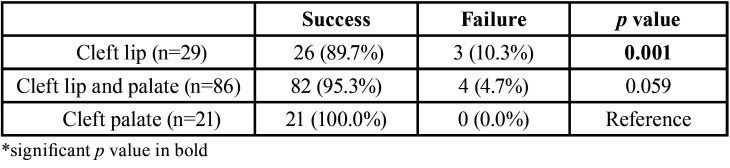
Success and failures rates of endodontic treatments according to the type of orofacial cleft.

**Figure 2:**
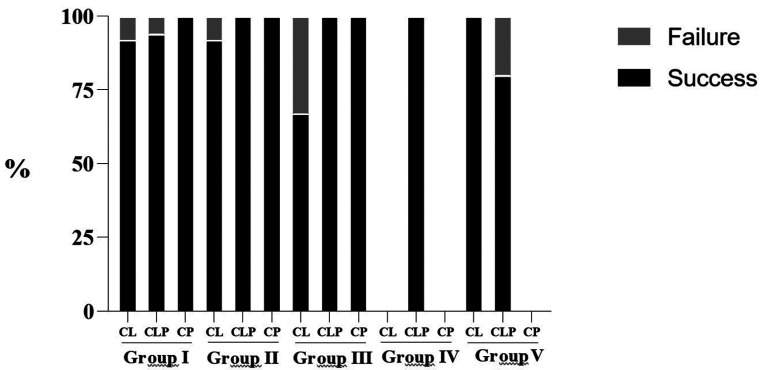
Success and failure rates of endodontic treatments according to the etiological factors and type of orofacial cleft.

Table 2 and Figure 2 present the correlation between the most prevalent etiological factors that led to the need of endodontic treatment, types of cleft and success and failure rates. Among the etiological factors observed, pulp involvement due to caries (Group I) was predominant (n=78); however, regardless of the type of cleft and cause for the indication of endodontic treatment, the percentage of success was higher in all cases, namely Group I - CP 100%, CLP 94.2% and CL 91.7%; Group II - CP 100%, CLP 100% and CL 92.3%; Group III - CP 100%, CLP 100% and CL 66.7%; Group IV - CLP 100% and Group V - CL 100% and CLP 80.0%.

**Table 2:**
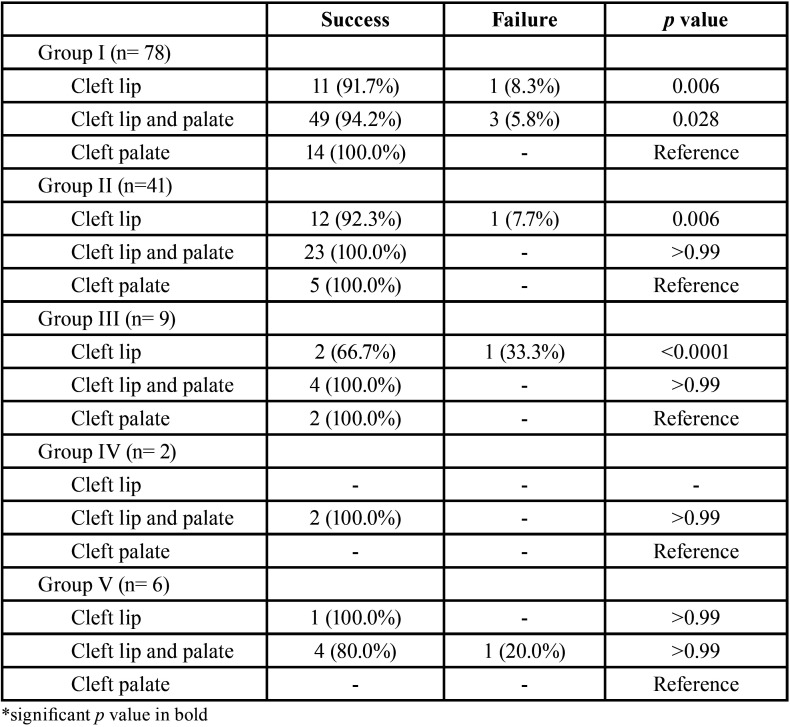
Success and failures rates of endodontic treatments according to the etiological factors and type of orofacial cleft.

Table 2 presents the comparison between groups, taking the cleft palate as reference, evidencing statistically significant difference for Group I between CP and CL (*P*=0.006) and between CP and CLP (*P*=0.028). In Group II, there was statistically significant difference (*P*=0.006) between CP and CL. In Group III, the difference was (*P*<0.0001) between CL and CP. There was no statistically significant difference in Groups IV and V, respectively.

## Discussion

Several factors are involved in the etiology of orofacial clefts, since any cause of physical, chemical or biological nature that can affect the differentiation, migration and proliferation of neural crest cells has the potential to determine the occurrence of clefts during embryonic development (7,8). In our study, Figure 1 shows that cleft lip and palate, which involves the lip, alveolar ridge and palate (CLP), was the most prevalent in all groups analyzed, since this cleft presents the greatest esthetic and functional impairment of the dental arches, impairing from breastfeeding to changes in maxillary growth, including dental anomalies and speech disorders (9). In addition, other data in the literature indicate cleft lip and palate as the most prevalent, mainly affecting the left side (9). This may also be explained by the higher rate of this type of cleft among individuals registered at the Hospital for Rehabilitation of Craniofacial Anomalies at the University of São Paulo (9). In the detailing of results, cleft palate (CP) showed a higher percentage regarding the incidence and treatment success; however, it should be mentioned that this type of cleft was used in the statistical analysis as a reference value, which established differences between some groups. Notwithstanding, we should be careful to note that the sample size (n) was smaller in this type of cleft compared to the other types included.

This study aimed to assess the etiological factors that led to the need of endodontic intervention; the patient’s medical history mentioned pulp involvement due to caries, indication of root canal treatment for prosthetic purposes, for orthodontic movement, tooth resorption, incompletely formed root, dental trauma and indication of internal tooth bleaching. Since the hygiene of individuals with orofacial clefts is usually deficient due to the abnormal dental arch morphology as a result of the congenital defect, they are susceptible to dental caries and all damages it causes to the oral environment. If not assisted by a dental professional to perform interventions, bacteria and their byproducts reach the pulp and the involved tooth should require endodontic treatment (1,6). This justifies the fact that pulp involvement due to caries (Group I) was observed as the cause with the highest incidence. The dental pulp, when subjected to various external stimuli, such as bacterial invasion, causes an inflammatory reaction in the tissues, resulting in clinical signs and symptoms of reversible and irreversible pulpitis, which can be “reversed” if the harmful stimulus is removed. However, if the stimulus exceeds the innate mechanisms of pulp defense and repair, it becomes painful (acute pain) and irreversible, resulting in pulp necrosis and requiring pulpectomy (10).

Due to the orofacial malformations, many patients require oral prosthetic rehabilitation to restore their masticatory and esthetic functions, and endodontic therapy for prosthetic purposes is a contributory step towards this objective. This study demonstrated that this was the second most prominent etiological factor (Group II) found in the studied population. In the presence of clefts with alveolar ridge involvement, rehabilitation can be performed by grinding the dental structure for fabrication of metallic or resin cores and fixed prostheses, requiring endodontic intervention in most cases (9,11).

During the treatment of individuals with clefts, surgical procedures as lip repair, palate repair, alveolar bone graft, orthognathic surgery, among others, are necessary for complete rehabilitation of the patient. Root resorption has been reported in some cases as a complication after these procedures, due to the injury suffered, especially in alveolar bone graft surgeries (9,12). Cases of tooth resorption may also be related to orthodontic movement during the treatment period in these individuals, since sometimes the force applied ultimately leads to the degeneration of cementoblasts. When the moved tooth compresses the periodontal ligament vessels in this region, these cells can necrotize or migrate (13). Based on the present results, orthodontic movement (Group IV) also appeared as an etiological factor leading to the need for endodontic intervention; however, it presented the lowest percentage of cases. Other authors justify these lower percentage data, pointing out that the pulp tissues do not undergo morphological and functional changes during orthodontic movement, regardless of the type and intensity of the force applied (13,14).

In this retrospective study, among the etiological factors, Group V comprised endodontic treatment endodontic (pulpectomy) for other causes, such as, dental trauma that resulted in pulp necrosis (15) and indication for internal tooth bleaching. Conversely, the intrinsic color changes are caused by several factors, which must be evidenced during clinical examination to allow greater predictability of results, making previous pulp therapy necessary. With the advances in Esthetic Dentistry, endogenous bleaching has proved to be a safe and less expensive alternative to restore the color harmony of non-vital teeth. The professional must use a clinical protocol based on scientific knowledge (16).

The absence of spontaneous and provoked painful symptoms, adequate sealing, tooth rehabilitated in masticatory function and the repair of apical and periapical tissues are clinical criteria for success in endodontic therapy. Cases of failure are closely related to the persistence of microorganisms after endodontic obturation or infection due to lack of coronal sealing. Thus, clinical and radiographic follow-up is important for the longevity of therapy; if any change is detected, endodontic reintervention is indicated, provided it can be performed. The European Society of Endodontics (2006) suggests the achievement of control radiographs for at least 1 year after completion of endodontic treatment (6,15,18,19). In cases of pulp necrosis with radiographically visible apical bone resorption, clinical and radiographic follow-up should be encouraged by the professional for a period of 2 to 4 years, due to the difficulty of performing histopathological analysis of the periapical lesion (6,20).

Regardless of the type of orofacial cleft and etiological factor involved, a high percentage of success was observed (Tables 1,2 and Fig. 2), which can be assigned to the knowledge of professionals and the technological and scientific advances in Endodontics in recent years, such as the use of digital radiography, cone-beam computed tomography, foraminal locators, engine-driven mechanic systems, use of ultrasound to enhance the properties of irrigants, intracanal medication and use of resin and bioceramic materials for root canal obturation (6,15,18,19,21). Therefore, this retrospective study revealed relevant data regarding the type of cleft and the most prevalent etiological factors that lead to the need for endodontic treatment. The highest ratio of treatment success evidences the importance of efficiency and quality of the care delivered to individuals with orofacial clefts.

## Conclusions

Cleft lip and palate affecting the lip, alveolar ridge and palate, as well as pulp involvement due to caries, were the most prevalent cleft type and etiological factor, respectively. The success of endodontic therapy prevailed in the procedures performed. These data are relevant, since the well-conducted root canal treatment is necessary for dental rehabilitation, maintaining the masticatory function and esthetic harmony of these individuals.

## References

[1] Allagh KP, Shamanna BR, Murthy GV, Ness AR, Doyle P, Neogi SB (2015). Birth prevalence of neural tube defects and orofacial clefts in India: a systematic review and meta-analysis. Plus One.

[2] Mutthineni RB, Nutalapati R, Kasagani SK (2010). Comparison of oral hygiene and periodontal status in patients with clefts of palate and patients with unilateral cleft lip, palate and alveolus. J Indian Soc Periodontol.

[3] Sooratgar A, Tabrizizade M, Nourelahi M, Asadi Y, Sooratgar H (2016). Management of an endodontic-periodontal lesion in a maxillary lateral incisor with palatal radicular groove: a case report. Iran Endod J.

[4] Uraba S, Ebihara A, Komatsu K, Ohbayashi N, Okiji T (2016). Ability of cone-beam computed tomography to detect periapical lesions that were not detected by periapical radiography: a retrospective assessment according to tooth group. J Endod.

[5] Demeter A, Bogdán S, Tóth Z, Nemes J (2014). Complex treatment of a large radicular cyst due to traumatic dental injury-a case report. Fogorv Sz.

[6] Santos-Junior AO, Pinto LC, Mateo-Castillo JF, Pinheiro CR (2019). Success or failure of endodontic treatments: a retrospective study: a retrospective study. J Conserv Dent.

[7] Murray JC (1995). Face facts: genes, environment and clefts. Am J Hum Genet.

[8] Vieira AR (2008). Unraveling human cleft lip and palate research. J Dent Res.

[9] Freitas JA, Neves LT, Almeida AL, Garib DG, Trindade-Suedam IK, Yaedo RY (2012). Rehabilitative treatment of cleft lip and palate: experience of the Hospital for Rehabilitation of Craniofacial Anomalies/USP (HRAC/USP) - Part 1: overall aspects. J Appl Oral Sci.

[10] Gemmell A, Stone S, Edwards D (2020). Investigating acute management of irreversible pulpitis: a survey of general dental practitioners in North East England. Br Dent J.

[11] Tavano RD, Balla MV, Leal CR, Lopes JF, Pinto JH (2015). Bite force in patients with cleft lip and palate with prosthetic rehabilitation. Rev Odontol Arac.

[12] Aguiar CR, Pinto LC, Nishiyama CK, Hussne RP (2014). Evaluation of the relationship between dental resorptions and cleft lip and/or palate, and its prevalence in patients treated at the department of endodontics, hospital for rehabilitation of craniofacial anomalies. ROBRAC.

[13] Consolaro A, Consolaro RB (2013). Orthodontic movement of endodontically treated teeth. Dental Press J Orthod.

[14] Hamilton RS, Gutmann JL (1999). Endodontic-orthodontic relationships: a review of integrated treatment planning challenges. Int Endod J.

[15] Fouad AF (2019). Microbiological Aspects of Traumatic Injuries. J Endod.

[16] Cardoso RM, Cardoso RM, Melo Júnior PC, Menezes PF Filho (2011). Intra coronal bleaching: an alternative to dyschromia of endodontically treated teeth: case report. Odontol Clín-Cient.

[17] Muliyar S, Shameem KA, Thankachan RP, Francis PG, Jayapalan CS, Hafiz KA (2014). Microleakage in endodontics. J Int Oral Health.

[18] Bergenholtz G (2016). Assessment of treatment failure in endodontic therapy. J Oral Rehabil.

[19] Abu Hasna A, Ungaro DM, Melo AA, Yui KC, Silva EG, Martinho FC (2019). Nonsurgical endodontic management of dens invaginatus: a report of two cases. F1000Res.

[20] Riyahi AM, Bashiri A, Alshahrani K, Alshahrani S, Alamri HM, Al-Sudani D (2020). Cyclic Fatigue Comparison of TruNatomy, Twisted File, and ProTaperNext Rotary Systems. Int J Dent.

[21] Alsubait S, Alsaad N, Alahmari S, Alfaraj F, Alfawaz H, Alqedairi A (2020). The effect of intracanal medicaments used in endodontics on the dislocation resistance of two calcium silicate-based filling materials. BMC Oral Health.

